# 4,4′-Bipyridine–3-(thio­phen-3-yl)acrylic acid (1/2)

**DOI:** 10.1107/S1600536811035823

**Published:** 2011-09-14

**Authors:** Palanisamy Rajakannu, Firasat Hussain, Malaichamy Sathiyendiran

**Affiliations:** a Department of Chemistry, University of Delhi, North Campus, Delhi, India

## Abstract

In the title 1/2 adduct, C_10_H_8_N_2_·2C_7_H_6_O_2_S, the dihedral angle between the pyridine rings is 18.41 (11)°. In the thio­phene­acrylic acid mol­ecules, the dihedral angles between the respective thio­phene and acrylic acid units are 5.52 (17)° and 23.92 (9)°. In the crystal, the components are linked *via* O—H⋯N hydrogen-bonding inter­actions, forming units of two 3-thio­phene­acrylic acid mol­ecules and one 4,4′-bipyridine mol­ecule.

## Related literature

For the synthesis and *in vitro* anti­bacterial activity of oxazolidines, see: Srivastava *et al.* (2008 ▶). For crystal engineering co-crystal and polymorph architectures, see: Friščić & MacGillivray (2009 ▶); Eccles *et al.* (2010 ▶). For the supra­molecular construction of mol­ecular ladders, see: Gao *et al.* (2004 ▶); MacGillivray *et al.* (2008 ▶); Friščić & MacGillivray (2005 ▶). For C—H⋯O hydrogen bonds in supra­molecular design, see: Desiraju (1996 ▶) and for C—H⋯π inter­actions in crystal engineering, see: Desiraju (2002 ▶). 
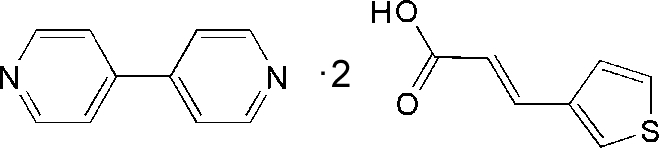


## Experimental

### 

#### Crystal data

C_10_H_8_N_2_·2C_7_H_6_O_2_S
*M* *_r_* = 464.54
Triclinic, 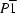

*a* = 7.3454 (5) Å
*b* = 10.7319 (8) Å
*c* = 15.0196 (11) Å
α = 102.518 (6)°
β = 103.648 (6)°
γ = 94.892 (6)°
*V* = 1111.54 (14) Å^3^
*Z* = 2
Cu *K*α radiation
μ = 2.46 mm^−1^
*T* = 293 K
0.37 × 0.15 × 0.10 mm

#### Data collection

Oxford Diffraction Xcalibur Sapphire3 diffractometer
Absorption correction: multi-scan (*CrysAlis PRO*; Oxford Diffraction, 2009 ▶) *T*_min_ = 0.692, *T*_max_ = 1.000
9038 measured reflections
4344 independent reflections
3498 reflections with *I* > 2σ(*I*)
*R*_int_ = 0.027

#### Refinement

*R*[*F* ^2^ > 2σ(*F* ^2^)] = 0.045
*wR*(*F* ^2^) = 0.132
*S* = 1.05
4344 reflections
291 parameters
H-atom parameters constrained
Δρ_max_ = 0.24 e Å^−3^
Δρ_min_ = −0.32 e Å^−3^

### 

Data collection: *CrysAlis PRO* (Oxford Diffraction 2009) ▶; cell refinement: *CrysAlis RED* (Oxford Diffraction, 2009) ▶; data reduction: *CrysAlis RED* ▶; program(s) used to solve structure: *SHELXS97* (Sheldrick, 2008 ▶); program(s) used to refine structure: *SHELXL97* (Sheldrick, 2008 ▶); molecular graphics: *ORTEP-3* (Farrugia, 1997 ▶); software used to prepare material for publication: *publCIF* (Westrip, 2010 ▶) and *PLATON* (Spek, 2009 ▶).

## Supplementary Material

Crystal structure: contains datablock(s) I, global. DOI: 10.1107/S1600536811035823/si2368sup1.cif

Structure factors: contains datablock(s) I. DOI: 10.1107/S1600536811035823/si2368Isup2.hkl

Supplementary material file. DOI: 10.1107/S1600536811035823/si2368Isup3.cml

Additional supplementary materials:  crystallographic information; 3D view; checkCIF report

## Figures and Tables

**Table 1:** Hydrogen-bond geometry (Å, °)

*D*—H⋯*A*	*D*—H	H⋯*A*	*D*⋯*A*	*D*—H⋯*A*
O2—H2⋯N1^i^	0.82	1.86	2.668 (2)	168
O4—H4*A*⋯N2^ii^	0.82	1.87	2.684 (2)	174

Symmetry codes: (i) 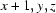; (ii) 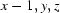.

**Fig. 1.**
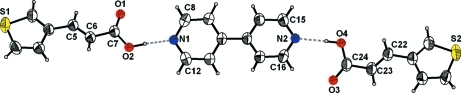
ORTEP view of the molecule with thermal ellipsoids drawn at 50% probability level Color code: White: C; red: O; blue: N; grey: H; yellow:S;

**Fig. 2.**
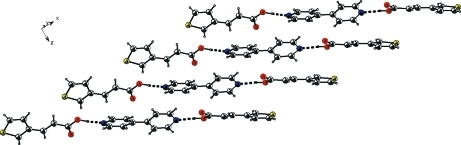
One-dimensional latterane like sheet formed though π-π stacking interactions between the two neighboring heterosynthons.

**Fig. 3.**
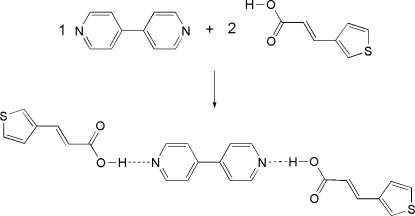
Synthesis of co-crystal of 4,4'-bipyridine and di(3-thiopheneacrylic acid)

## supplementary crystallographic information

### Comment

Supramolecular synthons that are based upon hydrogen bonds represent a prototypal tool for crystal engineering (Desiraju, 1996; 2002). Supramolecular heterosynthons formed from pyridine/amide and carboxylic acids have previously been exploited for liquid crystalline materials, two-dimensional beta networks, two-dimensional corrugated sheets and ternary supramolecules (MacGillivray *et al.*, 2008; Gao *et al.*, 2004; Friščić & MacGillivray, 2005; 2009). Recently, pharmaceutical molecules such as aspirin, *rac*-ibuprofen, and *rac*-flurbiprofen form heterosynthons with ditopic pyridine donors. Herein, we report co-crystal 1 synthesized and characterized by FT—IR, UV-Vis, ^1^H-NMR spectroscopy, EA, DSC, and TGA.

The co-crystal 1 of the 2:1 adduct of 3-thiopheneacrylic acid with 4,4'-bipyridine was obtained by layering methanolic solution of 4,4'-bipyridyl to the methanolic solution of 3-thiopheneacrylic acid at room temperature. Each 3-thiopheneacrylic acid molecule forms a moderate intermolecular O—H···N bond with pyridine (Table 1). The 4,4'-bipyridine molecule in the adduct is non-planar with the two pyridine rings forming a dihedral angle of 18.41 (11)°. The two thiophene and the bipyridine are not coplanar and the dihedral angles between the S1 thiophene/N1 pyridine and S2 thiophene/ N2 pyridine are 30.14 (11)° and 47.64 (7)°, respectively. The heterosynthon extends to one-dimensional latterane like sheets held together by moderate π-π stacking interactions (Fig. 2). The *Cg*1–*Cg*2^ii^ distance (between the N1,C8-C12 and N2,C13-C17 4,4'-bipyridine moieties) and the dihedral angle between pyridine planes *α* are 4.1411 (13)Å and 18.4 (1)°, respectively. [Symmetry code ii: (-1+*x*,y,*z*).

### Experimental

All starting materials and products were found to be stable towards moisture and air. Starting materials such as 4,4'-bipyridyl (bpy) and 3-thiopheneacrylic acid (taa) were procured from commercial sources and used as received. Commercial grade solvents *e.g.* methanol was used as received further purification. The mixture of 1:2 ratio of 4,4'-bipyridyl (100.1 mg, 0.6409 mmol) and 3-thiopheneacrylic acid (197.8 mg, 1.2828 mmol) in methanol was stirred for 3 h at room temperature. The clear solution was obtained by filtration and that solution was kept at room temperature for several days. The white colored crystals were obtained. Yield: 83% (248.3 mg, 0.5344 mmol). Anal. Calcd for C_24_H_20_N_2_O_4_S_2_: C, 62.05; H, 4.34; N, 6.03; S, 13.8. Found: C, 60.93; H, 4.13; N, 5.87; S, 12.93. ^1^H NMR (CDCl_3_,): 8.72 (dd, *J* = 4.7 Hz, 4H, H^α^, bpy), 7.71 (d, *J* = 1.54 Hz, 2H, H^4^, taa), 7.53 (dd, *J* = 4.7 Hz, 4H, H^β^, bpy), 7.47 (dd, *J* = 1.32 Hz, 2H, H^1^, taa), 7.29 (m, 4H, H^2,3^, taa), 6.20 (d, *J* = 15.44 Hz, 2H, H^5^, taa).

### Refinement

All H atoms were placed in geometrically calculated positions and refined using a riding model, with C—H = 0.95–1.00 Å and *U*_iso_(H) = 1.2Ueq(C).

### Crystal data

**Table:**

C_10_H_8_N_2_·2C_7_H_6_O_2_S	*Z* = 2
*M**_r_* = 464.54	*F*(000) = 484
Triclinic, *P*1	*D*_x_ = 1.388 Mg m^−^^3^
Hall symbol: -P 1	Cu *K*α radiation, λ = 1.54184 Å
*a* = 7.3454 (5) Å	Cell parameters from 3251 reflections
*b* = 10.7319 (8) Å	θ = 3.1–72.9°
*c* = 15.0196 (11) Å	µ = 2.46 mm^−^^1^
α = 102.518 (6)°	*T* = 293 K
β = 103.648 (6)°	Plate, white
γ = 94.892 (6)°	0.37 × 0.15 × 0.10 mm
*V* = 1111.54 (14) Å^3^	

### Data collection

**Table:**

Xcalibur, Sapphire3 diffractometer	4344 independent reflections
Radiation source: fine-focus sealed tube	3498 reflections with *I* > 2σ(*I*)
graphite	*R*_int_ = 0.027
Detector resolution: 15.9853 pixels mm^-1^	θ_max_ = 72.1°, θ_min_ = 3.1°
ω scans	*h* = −8→9
Absorption correction: multi-scan (*CrysAlis PRO*; Oxford Diffraction, 2009)	*k* = −13→12
*T*_min_ = 0.692, *T*_max_ = 1.000	*l* = −18→13
9038 measured reflections	

### Refinement

**Table:**

Refinement on *F*^2^	Primary atom site location: structure-invariant direct methods
Least-squares matrix: full	Secondary atom site location: difference Fourier map
*R*[*F*^2^ > 2σ(*F*^2^)] = 0.045	Hydrogen site location: inferred from neighbouring sites
*wR*(*F*^2^) = 0.132	H-atom parameters constrained
*S* = 1.05	*w* = 1/[σ^2^(*F*_o_^2^) + (0.0658*P*)^2^ + 0.2003*P*] where *P* = (*F*_o_^2^ + 2*F*_c_^2^)/3
4344 reflections	(Δ/σ)_max_ < 0.001
291 parameters	Δρ_max_ = 0.24 e Å^−^^3^
0 restraints	Δρ_min_ = −0.32 e Å^−^^3^

### Special details

**Table:**

Geometry. All e.s.d.'s (except the e.s.d. in the dihedral angle between two l.s. planes) are estimated using the full covariance matrix. The cell e.s.d.'s are taken into account individually in the estimation of e.s.d.'s in distances, angles and torsion angles; correlations between e.s.d.'s in cell parameters are only used when they are defined by crystal symmetry. An approximate (isotropic) treatment of cell e.s.d.'s is used for estimating e.s.d.'s involving l.s. planes.
Refinement. Refinement of *F*^2^ against ALL reflections. The weighted *R*-factor *wR* and goodness of fit *S* are based on *F*^2^, conventional *R*-factors *R* are based on *F*, with *F* set to zero for negative *F*^2^. The threshold expression of *F*^2^ > σ(*F*^2^) is used only for calculating *R*-factors(gt) *etc*. and is not relevant to the choice of reflections for refinement. *R*-factors based on *F*^2^ are statistically about twice as large as those based on *F*, and *R*- factors based on ALL data will be even larger.

### Fractional atomic coordinates and isotropic or equivalent isotropic displacement parameters (Å^2^)

**Table:**

	*x*	*y*	*z*	*U*_iso_*/*U*_eq_	
C1	0.0548 (3)	0.0002 (2)	−0.17354 (15)	0.0547 (5)	
H1	0.0689	−0.0528	−0.1316	0.066*	
C2	0.1988 (3)	0.08388 (19)	−0.17968 (13)	0.0439 (4)	
C3	0.1338 (3)	0.1521 (2)	−0.25031 (15)	0.0542 (5)	
H3	0.2120	0.2136	−0.2645	0.065*	
C4	−0.0525 (3)	0.1183 (2)	−0.29432 (16)	0.0611 (6)	
H4	−0.1172	0.1531	−0.3420	0.073*	
C5	0.3899 (3)	0.09887 (19)	−0.12014 (13)	0.0447 (4)	
H5	0.4097	0.0504	−0.0753	0.054*	
C6	0.5392 (3)	0.1746 (2)	−0.12320 (14)	0.0479 (4)	
H6	0.5262	0.2230	−0.1681	0.058*	
C7	0.7262 (3)	0.18356 (19)	−0.05652 (14)	0.0467 (4)	
C8	0.2956 (3)	0.2219 (2)	0.08555 (15)	0.0559 (5)	
H8	0.2325	0.1394	0.0762	0.067*	
C9	0.4852 (3)	0.2485 (2)	0.13424 (15)	0.0508 (5)	
H9	0.5459	0.1850	0.1574	0.061*	
C10	0.5840 (3)	0.36980 (18)	0.14838 (13)	0.0429 (4)	
C11	0.4821 (3)	0.4604 (2)	0.11311 (16)	0.0571 (5)	
H11	0.5412	0.5437	0.1213	0.069*	
C12	0.2929 (3)	0.4253 (2)	0.06605 (17)	0.0610 (6)	
H12	0.2273	0.4872	0.0432	0.073*	
C13	0.7889 (3)	0.40367 (18)	0.19703 (13)	0.0430 (4)	
C14	0.8822 (3)	0.3308 (2)	0.25474 (15)	0.0537 (5)	
H14	0.8163	0.2593	0.2646	0.064*	
C15	1.0737 (3)	0.3656 (2)	0.29725 (16)	0.0575 (5)	
H15	1.1340	0.3149	0.3347	0.069*	
C16	1.0874 (3)	0.5381 (2)	0.23340 (17)	0.0595 (6)	
H16	1.1565	0.6104	0.2263	0.071*	
C17	0.8972 (3)	0.5096 (2)	0.18718 (16)	0.0556 (5)	
H17	0.8417	0.5614	0.1494	0.067*	
C18	1.2549 (3)	0.6734 (2)	0.52895 (16)	0.0584 (5)	
H18	1.2472	0.5856	0.5026	0.070*	
C19	1.1052 (3)	0.7401 (2)	0.51574 (13)	0.0475 (4)	
C20	1.1585 (3)	0.8722 (2)	0.56515 (17)	0.0614 (6)	
H20	1.0746	0.9324	0.5652	0.074*	
C21	1.3461 (3)	0.9011 (2)	0.61250 (18)	0.0660 (6)	
H21	1.4053	0.9830	0.6477	0.079*	
C22	0.9150 (3)	0.6807 (2)	0.46025 (13)	0.0490 (5)	
H22	0.8956	0.5918	0.4362	0.059*	
C23	0.7683 (3)	0.7418 (2)	0.44109 (15)	0.0548 (5)	
H23	0.7857	0.8303	0.4668	0.066*	
C24	0.5778 (3)	0.6803 (2)	0.38173 (15)	0.0549 (5)	
O1	0.7640 (2)	0.11251 (16)	−0.00462 (11)	0.0637 (4)	
O2	0.8489 (2)	0.27837 (16)	−0.05983 (12)	0.0645 (4)	
H2	0.9510	0.2794	−0.0227	0.097*	
O3	0.4670 (2)	0.74129 (19)	0.34314 (14)	0.0809 (6)	
O4	0.5423 (2)	0.55602 (16)	0.37539 (13)	0.0650 (4)	
H4A	0.4325	0.5289	0.3450	0.098*	
S1	−0.15372 (8)	0.00362 (7)	−0.25123 (4)	0.0671 (2)	
S2	1.45740 (8)	0.76787 (7)	0.59882 (5)	0.0697 (2)	
N1	0.1992 (2)	0.30804 (19)	0.05151 (13)	0.0568 (5)	
N2	1.1767 (2)	0.46765 (18)	0.28753 (13)	0.0552 (4)	

### Atomic displacement parameters (Å^2^)

**Table:**

	*U*^11^	*U*^22^	*U*^33^	*U*^12^	*U*^13^	*U*^23^
C1	0.0390 (10)	0.0657 (13)	0.0557 (12)	0.0000 (9)	0.0023 (8)	0.0207 (10)
C2	0.0382 (10)	0.0472 (10)	0.0429 (9)	0.0050 (8)	0.0058 (7)	0.0098 (8)
C3	0.0474 (11)	0.0583 (12)	0.0553 (12)	0.0055 (9)	0.0045 (9)	0.0213 (10)
C4	0.0512 (12)	0.0715 (14)	0.0548 (12)	0.0142 (11)	−0.0025 (9)	0.0193 (11)
C5	0.0378 (10)	0.0497 (10)	0.0448 (10)	0.0062 (8)	0.0048 (8)	0.0147 (8)
C6	0.0390 (10)	0.0518 (11)	0.0511 (11)	0.0052 (8)	0.0018 (8)	0.0200 (9)
C7	0.0358 (9)	0.0512 (11)	0.0511 (10)	0.0048 (8)	0.0049 (8)	0.0163 (9)
C8	0.0430 (11)	0.0567 (12)	0.0597 (12)	−0.0069 (9)	0.0073 (9)	0.0086 (10)
C9	0.0421 (10)	0.0491 (11)	0.0569 (11)	0.0014 (8)	0.0065 (9)	0.0130 (9)
C10	0.0351 (9)	0.0489 (10)	0.0400 (9)	0.0028 (8)	0.0049 (7)	0.0074 (8)
C11	0.0437 (11)	0.0502 (11)	0.0701 (13)	0.0020 (9)	−0.0004 (10)	0.0178 (10)
C12	0.0426 (11)	0.0653 (14)	0.0701 (14)	0.0101 (10)	0.0000 (10)	0.0208 (11)
C13	0.0338 (9)	0.0472 (10)	0.0422 (9)	0.0030 (7)	0.0039 (7)	0.0065 (8)
C14	0.0396 (10)	0.0610 (12)	0.0590 (12)	0.0026 (9)	0.0047 (9)	0.0224 (10)
C15	0.0415 (11)	0.0663 (14)	0.0609 (13)	0.0078 (10)	0.0005 (9)	0.0220 (11)
C16	0.0406 (11)	0.0591 (13)	0.0695 (14)	−0.0079 (9)	0.0010 (10)	0.0165 (11)
C17	0.0422 (11)	0.0544 (12)	0.0629 (13)	−0.0014 (9)	−0.0013 (9)	0.0186 (10)
C18	0.0406 (11)	0.0641 (13)	0.0612 (13)	0.0030 (9)	0.0058 (9)	0.0059 (10)
C19	0.0372 (10)	0.0588 (12)	0.0421 (9)	0.0004 (8)	0.0040 (7)	0.0121 (9)
C20	0.0439 (12)	0.0593 (13)	0.0700 (14)	0.0058 (10)	−0.0008 (10)	0.0101 (11)
C21	0.0461 (12)	0.0624 (14)	0.0716 (15)	−0.0046 (10)	−0.0014 (10)	0.0021 (11)
C22	0.0390 (10)	0.0576 (12)	0.0453 (10)	−0.0037 (9)	0.0036 (8)	0.0140 (9)
C23	0.0406 (11)	0.0612 (13)	0.0559 (12)	−0.0025 (9)	0.0000 (9)	0.0182 (10)
C24	0.0371 (10)	0.0682 (14)	0.0571 (12)	−0.0024 (9)	0.0027 (9)	0.0248 (10)
O1	0.0460 (8)	0.0779 (11)	0.0686 (10)	0.0059 (7)	−0.0012 (7)	0.0399 (9)
O2	0.0389 (8)	0.0669 (10)	0.0806 (11)	−0.0046 (7)	−0.0068 (7)	0.0324 (8)
O3	0.0477 (9)	0.0871 (12)	0.0997 (13)	−0.0059 (9)	−0.0160 (9)	0.0502 (11)
O4	0.0359 (8)	0.0668 (10)	0.0792 (11)	−0.0008 (7)	−0.0064 (7)	0.0169 (8)
S1	0.0359 (3)	0.0853 (4)	0.0685 (4)	−0.0024 (3)	−0.0014 (2)	0.0156 (3)
S2	0.0343 (3)	0.0880 (5)	0.0729 (4)	0.0059 (3)	−0.0003 (2)	0.0073 (3)
N1	0.0350 (9)	0.0705 (12)	0.0563 (10)	−0.0002 (8)	0.0024 (7)	0.0110 (9)
N2	0.0359 (9)	0.0640 (11)	0.0558 (10)	0.0012 (8)	0.0006 (7)	0.0089 (8)

### Geometric parameters (Å, °)

**Table:**

C1—C2	1.361 (3)	C13—C14	1.392 (3)
C1—S1	1.701 (2)	C14—C15	1.382 (3)
C1—H1	0.9300	C14—H14	0.9300
C2—C3	1.430 (3)	C15—N2	1.332 (3)
C2—C5	1.451 (3)	C15—H15	0.9300
C3—C4	1.351 (3)	C16—N2	1.328 (3)
C3—H3	0.9300	C16—C17	1.380 (3)
C4—S1	1.703 (3)	C16—H16	0.9300
C4—H4	0.9300	C17—H17	0.9300
C5—C6	1.324 (3)	C18—C19	1.362 (3)
C5—H5	0.9300	C18—S2	1.699 (2)
C6—C7	1.478 (3)	C18—H18	0.9300
C6—H6	0.9300	C19—C20	1.423 (3)
C7—O1	1.207 (2)	C19—C22	1.456 (3)
C7—O2	1.318 (2)	C20—C21	1.367 (3)
C8—N1	1.328 (3)	C20—H20	0.9300
C8—C9	1.385 (3)	C21—S2	1.705 (3)
C8—H8	0.9300	C21—H21	0.9300
C9—C10	1.383 (3)	C22—C23	1.315 (3)
C9—H9	0.9300	C22—H22	0.9300
C10—C11	1.395 (3)	C23—C24	1.479 (3)
C10—C13	1.485 (2)	C23—H23	0.9300
C11—C12	1.380 (3)	C24—O3	1.208 (3)
C11—H11	0.9300	C24—O4	1.315 (3)
C12—N1	1.330 (3)	O2—H2	0.8200
C12—H12	0.9300	O4—H4A	0.8200
C13—C17	1.386 (3)		
			
C2—C1—S1	112.31 (16)	C15—C14—C13	119.4 (2)
C2—C1—H1	123.8	C15—C14—H14	120.3
S1—C1—H1	123.8	C13—C14—H14	120.3
C1—C2—C3	110.97 (18)	N2—C15—C14	123.6 (2)
C1—C2—C5	122.51 (18)	N2—C15—H15	118.2
C3—C2—C5	126.52 (18)	C14—C15—H15	118.2
C4—C3—C2	113.3 (2)	N2—C16—C17	123.3 (2)
C4—C3—H3	123.4	N2—C16—H16	118.3
C2—C3—H3	123.4	C17—C16—H16	118.3
C3—C4—S1	111.33 (17)	C16—C17—C13	120.1 (2)
C3—C4—H4	124.3	C16—C17—H17	120.0
S1—C4—H4	124.3	C13—C17—H17	120.0
C6—C5—C2	126.60 (18)	C19—C18—S2	112.62 (18)
C6—C5—H5	116.7	C19—C18—H18	123.7
C2—C5—H5	116.7	S2—C18—H18	123.7
C5—C6—C7	121.21 (18)	C18—C19—C20	111.27 (19)
C5—C6—H6	119.4	C18—C19—C22	123.4 (2)
C7—C6—H6	119.4	C20—C19—C22	125.35 (19)
O1—C7—O2	123.23 (18)	C21—C20—C19	112.8 (2)
O1—C7—C6	124.35 (18)	C21—C20—H20	123.6
O2—C7—C6	112.42 (17)	C19—C20—H20	123.6
N1—C8—C9	123.5 (2)	C20—C21—S2	111.29 (18)
N1—C8—H8	118.3	C20—C21—H21	124.4
C9—C8—H8	118.3	S2—C21—H21	124.4
C10—C9—C8	119.7 (2)	C23—C22—C19	125.7 (2)
C10—C9—H9	120.1	C23—C22—H22	117.1
C8—C9—H9	120.1	C19—C22—H22	117.1
C9—C10—C11	116.67 (18)	C22—C23—C24	124.9 (2)
C9—C10—C13	122.63 (18)	C22—C23—H23	117.6
C11—C10—C13	120.69 (18)	C24—C23—H23	117.6
C12—C11—C10	119.5 (2)	O3—C24—O4	124.2 (2)
C12—C11—H11	120.3	O3—C24—C23	121.6 (2)
C10—C11—H11	120.2	O4—C24—C23	114.25 (18)
N1—C12—C11	123.6 (2)	C7—O2—H2	109.5
N1—C12—H12	118.2	C24—O4—H4A	109.5
C11—C12—H12	118.2	C1—S1—C4	92.12 (11)
C17—C13—C14	116.54 (18)	C18—S2—C21	91.96 (11)
C17—C13—C10	121.64 (18)	C8—N1—C12	117.01 (18)
C14—C13—C10	121.82 (18)	C16—N2—C15	117.01 (18)

### Hydrogen-bond geometry (Å, °)

**Table:**

*D*—H···*A*	*D*—H	H···*A*	*D*···*A*	*D*—H···*A*
O2—H2···N1^i^	0.82	1.86	2.668 (2)	168.
O4—H4A···N2^ii^	0.82	1.87	2.684 (2)	174.

Symmetry codes: (i) *x*+1, *y*, *z*; (ii) *x*−1, *y*, *z*.
